# Low-Birth-Weight among Term Newborns Born to Anaemic Pregnant Women Admitted to the Department of Obstetrics and Gynecology in a Tertiary Care Centre

**DOI:** 10.31729/jnma.8262

**Published:** 2023-09-30

**Authors:** Priya Yadav, Priyanka Yadav, Rakesh Kumar Das, Ram Das, Ashish Bhattarai, Saroj Shrestha

**Affiliations:** 1 Nepalgunj Medical College and Teaching Hospital, Kohalpur, Banke, Nepal; 2 Department of Obstetrics and Gynecology, Nepal Medical College and Teaching Hospital, Jorpati, Kathmandu, Nepal; 3 Department of Pediatrics, Lincoln Medical and Mental Health Center, Bronx, New York, USA

**Keywords:** *anaemia*, *infant*, *low birth weight*, *morbidity*, *pregnancy*

## Abstract

**Introduction:**

Anaemia is one of the most common conditions which affects a significant proportion of pregnant women worldwide. These patients may have adverse effects on both the mother and the developing fetus. Detecting and timely treating anaemia in pregnancy help in the overall improvement of maternal and fetal health. The aim of the study was to find out the prevalence of low-birth-weight among term newborns born to anaemic pregnant women admitted to the Department of Obstetrics and Gynecology in a tertiary care centre.

**Methods:**

A descriptive cross-sectional study was conducted among pregnant women who were diagnosed with anaemia and admitted for delivery in the Department of Obstetrics and Gynecology after obtaining ethical approval from the Institutional Review Committee. Data was collected from 10 December 2022 to 10 March 2023. Convenience sampling method was used. The point estimate was calculated at a 95% Confidence Interval.

**Results:**

Among 300 newborns, the prevalence of low-birth-weight was 106 (35.33%) (29.92-40.74, 95% Confidence Interval). Among 106 newborn, 64 (60.37%) were male and 42 (39.62%) were female.

**Conclusions:**

The prevalence of low-birth-weight among newborns born to term anaemic pregnant women admitted to the Department of Obstetrics and Gynecology in a tertiary care centre was found to be higher than in studies done in a similar settings.

## INTRODUCTION

Anaemia is one of the most frequently observed nutritional deficiency diseases in the world. During pregnancy iron deficiency anemia is often a contributory cause of maternal death.^1^ World Health Organization (WHO) defines anaemia in pregnancy as Hemoglobin less than 11gm/dl.^2^ Low Birth Weight (LBW) has been defined as a birth weight of less than 2,500g.^3^

Maternal anaemia is one of the important gestational outcomes for LBW and is considered a major public health problem which is more prevalent in countries with fewer financial resources.^4^ Maternal anaemia demands attention, not only because it affects the mother's health condition but also because it is related to undesirable gestational outcomes including infant mortality and morbidity.^5^

The aim of the study was to find out the prevalence of low-birth-weight among term newborns born in term anaemic pregnant women admitted to the Department of Obstetrics and Gynecology in a tertiary care centre.

## METHODS

A descriptive cross-sectional study was conducted in Nepalgunj Medical College and Teaching Hospital, Kohalpur, Banke, Nepal after receiving the ethical approval from the Institutional Review Committee (Reference number: 37/079-080) where data were collected from pregnant women who were diagnosed with anaemia and admitted for delivery from 10 December 2022 to 10 March 2023. All the newborn born after the period of gestation of ≥37 weeks from anaemic pregnant women admitted to the Department of Obstetrics and Gynecology in the tertiary care centre up to the study period were included in the study after consent from their mothers. The exclusion criteria included women who did not give consent to the study. Convenience sampling method was used. The sample size was calculated by using the following formula:


$$\begin{array}{ll} \text{n} & ={\text{Z}}^{2}\times \frac{\text{p}\times \text{q}}{{\text{e}}^{\text{2}}}\\ & =1.96^{2}\times \frac{0.179\times 0.821}{0.05^{2}}\\ & =226 \end{array}$$

Where,

n = minimum required sample size
Z = 1.96 for 95% Confidence Interval (CI)
p = prevalence of low-birth-weight taken from previous study as, 17.9 %^6^
q = 1-p
e = margin of error, 5%

The minimum required sample size was 226. However, the final sample size taken was 300.

Data were collected from the preformed questionnaire and low birth weight was diagnosed from WHO operational definition.^3^ World Health Organization (WHO) defines anemia in pregnancy as Hemoglobin less than 11gm/dl.^2^

Data was entered in Microsoft Excel 2016 and analysed using IBM SPSS Statistics version 26.0. The point estimate was calculated at a 95% CI.

## RESULTS

**Figure 1:**
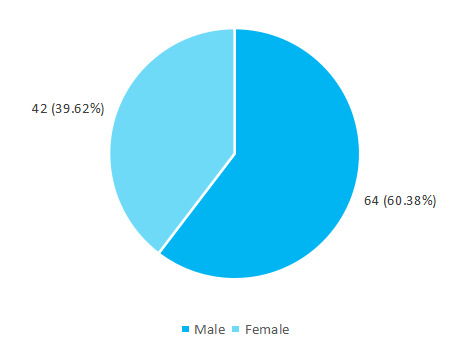
Gender-wise distribution of newborn with low-birth-weight (n= 106).

Among 300 term newborns born to anaemic pregnant women, the prevalence of low-birth-weight was 106 (35.33%) (29.92-40.74, 95% CI). Among 106 newborn, 64 (60.38%) were male (Figure 1).

**Figure 2:**
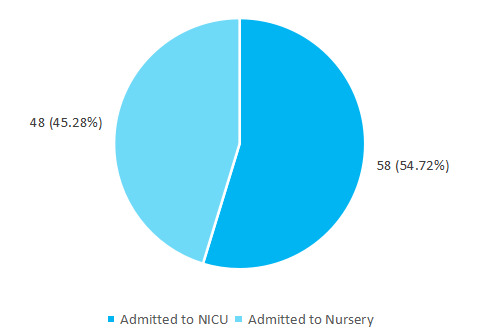
Admission of the newborn with low-birth-weight (n= 106).

Among newborn with low-birth-weight, 58 (54.72%) were admitted to the neonatal intensive care unit (NICU) (Figure 2).

## DISCUSSION

The prevalence of low-birth-weight was found to be 106 (35.33%) which is higher than other similar studies. WHO estimates that 40% of pregnant women worldwide are anaemic.^7^ One of the studies showed that higher rates of LBW were found among patients with anaemia as compared to non-anaemic women (10.5% versus 9.4%).^8^ Our study points out that a higher percentage of newborn who were LBW were male 64 (60.37%) compared to female 42 (39.62%) which is different from the study conducted in Sub-Saharan Africa which shows no significant difference between the genders.^9^

Our study shows that 58 (54.71%) low-birth-weight were admitted to the NICU. The incidence of low birth weight increases 2 fold and perinatal mortality by two to threefold when maternal haemoglobin is less than 8 gm%.^10^ Approximately 7-15% of all live births each year are of low birth weight.^11^

The study has few limitations as the study is done in a single medical institution and in a single nation, so this research cannot be generalized to all the other places. Also, since this is a descriptive cross-sectional study so the causal relation of maternal anaemia with other variables cannot be identified through this research. We have the potential bias for the missing data so this might influence the overall result of this research.

## CONCLUSIONS

The prevalence of low-birth-weight among term newborns born to anaemic pregnant women in our study was higher than in the studies done in similar settings. Efforts should be directed towards early identification, prevention, and effective management of anaemia during pregnancy to improve maternal and neonatal health outcomes.
